# Microbiological investigation of pregnancies following vaginal radical trachelectomy using 16S rRNA sequencing of FFPE placental specimens

**DOI:** 10.1002/2211-5463.13892

**Published:** 2024-09-08

**Authors:** Risa Tsunematsu, Tasuku Mariya, Mina Umemoto, Shiori Ogawa, Wataru Arai, Suguru E. Tanaka, Kyota Ashikawa, Terufumi Kubo, Yoshiyuki Sakuraba, Tsuyoshi Baba, Shinichi Ishioka, Toshiaki Endo, Tsuyoshi Saito

**Affiliations:** ^1^ Department of Obstetrics and Gynecology Sapporo Medical University School of Medicine Sapporo Japan; ^2^ Varinos Inc. Tokyo Japan; ^3^ Department of Pathology 1st Sapporo Medical University School of Medicine Sapporo Japan

**Keywords:** 16S rRNA sequencing, chorioamnionitis, formalin‐fixed paraffin‐embedded specimen, placenta, vaginal radical trachelectomy

## Abstract

This study examined the risk of intrauterine infection associated with radical trachelectomy (RT) in early‐stage cervical cancer patients. This procedure preserves fertility but is linked to increased risk of intrauterine infection due to cervical defects during pregnancy. DNA was extracted from the formalin‐fixed paraffin‐embedded (FFPE) placental specimens of 23 pregnant post‐RT patients and 16S rRNA gene sequencing was used for bacterial identification. The prevalence of *Lactobacillus crispatus* and *Burkholderia stabilis* was significantly higher in the non‐chorioamnionitis group. In contrast, alpha diversity analysis using the PD index showed significantly higher diversity in the chorioamnionitis group (*P* = 0.04). The demonstrated relationship between chorioamnionitis and microbial diversity affirms the importance of controlling the genital bacterial flora in pregnancies following RT.

## Graphical abstract

The study investigates the risk of intrauterine infections during pregnancy following radical trachelectomy (RT) in early‐stage cervical cancer patients. Using 16S rRNA sequencing on FFPE placental specimens, the research highlights a correlation between microbial diversity and chorioamnionitis, emphasizing the need for careful management of genital bacterial flora in pregnancies post‐RT.

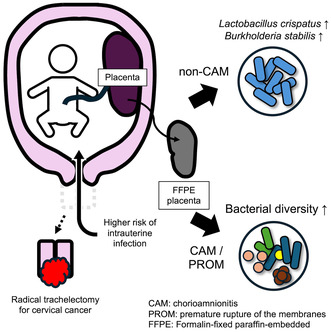

**Abbreviations**
CAM: Chorioamnionitis
FFPE: Formalin‐fixed paraffin‐embedded
PI: Placental infarction
RT: Radical trachelectomy
VRT: Vaginal radical trachelectomy

Cervical cancer often affects young women of reproductive age, and fertility cannot be preserved in treatment for advanced disease. Therefore, cervical cancer prevention through human papilloma virus (HPV) vaccination and early detection and treatment through HPV and cytological screening are crucial [1, 2, 3].

We previously attempted fertility preservation by performing vaginal radical trachelectomy (VRT) in 71 women with International Federation of Gynecology and Obstetrics (FIGO) 2009 stage 1A2‐1B1 uterine cervical cancer and achieved 24 live births in 28 pregnancies [4]. However, we have also experienced many pregnancy complications after radical trachelectomy (RT) [5]. Cervical conization is chosen for cervical intraepithelial neoplasia or early‐stage cervical cancers. However, this procedure can increase the risk of preterm birth and premature rupture of membranes (PROM) due to cervical shortening [6]. Compared with cervical conization, RT results in a larger defect of the cervix, which can have a significant impact on prenatal outcomes. While we have attempted measures such as frequent vaginal disinfection with povidone‐iodine and prophylactic antibiotic administration to prevent ascending infections, it appears that the increased risk of infection due to the large cervical defect remains considerable [5]. Furthermore, as a surgical approach, we have attempted preventative transabdominal cerclage (TAC) to treat cervical incompetence after RT [7, 8]. While a surgical procedure is necessary to remove the cerclage thread, the effectiveness of TAC for cervical incompetence has been clearly reported [9]. We believe that TAC after RT should be actively considered, depending on the extent of the cervical defect by surgical procedure.

In the post‐RT clinical course, it can be difficult to obtain effective intrauterine culture results. This is because results from a pregnant uterus can only be acquired via invasive tests, such as amniocentesis, which itself carries a risk of preterm birth and preterm rupture of membranes (PROM). One study demonstrated the usefulness of amniocentesis in cases of threatened preterm birth and antibiotic treatment [10]. However, this test might not be actively performed in high‐risk patients such as post‐RT patients. Furthermore, even if successful sample collection is achieved during a cesarean section, many vaginal bacteria, including anaerobic bacteria that would be able to cause ascending infection from the vagina, are challenging to culture [11, 12]. In recent years, reports have suggested the potential for bacterial detection and microbiome analysis in formalin‐fixed paraffin‐embedded (FFPE) tissue specimens through 16S rRNA analysis [13]. Indeed, we have previously reported the retrospective detection of invasive *Streptococcus pyogenes* infection from FFPE placental specimens [14]. Based on our experience, 16S rRNA analysis of FFPE specimens is challenging due to the difficulty of excluding contamination from environmental bacteria. Nevertheless, if universal FFPE specimens can be retrospectively analyzed to detect bacterial infection or analyze the microbiome, significant advances can be expected.

In this study, we retrospectively performed 16S rRNA analysis to determine the presence of intrauterine infection and identified specific causative bacteria in placental specimens obtained from pregnant women who had undergone RT.

## Materials and methods

### Ethical approval and consent to participate

In our study, informed consent was obtained through an opt‐out procedure on the Sapporo Medical University's website. This opt‐out consent process allowing individuals to express their preference not to participate in anytime they hope. This study was approved by the Ethics Committee of Sapporo Medical University (No. 312–63) and the study was conducted in accordance with the guidelines set by the Declaration of Helsinki.

### PCR amplification, library preparation, and sequencing of FFPE placental samples

Placentas were obtained from 23 post‐RT women after delivery at Sapporo Medical University. FFPE placental specimens were then prepared according to standard methods (Table 1). A case of term delivery without any obvious intrauterine infection at 40 weeks of gestation was used as a negative infection control. Each FFPE block of a placental specimen was sliced into 10‐μm‐thick sections. DNA was extracted from the 40‐mg sections using a Qiagen GeneRead DNA FFPE kit (Qiagen, Hilden, Germany) according to the manufacturer's instructions. The extracted DNA concentration was quantified by fluorescence measurement with a Qubit fluorometer (Thermo Fisher Scientific, Waltham, MA, USA).

**Table 1:** Clinical background and placental findings in cases of miscarriage or delivery after radical trachelectomy. CAM, Chorioamnionitis; LsRT, Laparoscopic semiradical trachelectomy; NA, Not assessed; PLN, Pelvic lymph node adenectomy; VRT, Vaginal radical trachelectomy.

No.	Age	Gravida	Parous	Stage	Surgery	Histological diagnosis of cancer	ART for infertility	Causes of termination	Delivery	Birth weight (g)	Placental weight (g)	Histological findings in placenta (Blanc CAM staging)
1	33	0	0	IB1	VRT + PLN	Adenosquamous		PROM	32w0d	NA	NA	Placental infarction
2	39	0	0	IB1	VRT + PLN	Adenosquamous		PROM	26w1d	878	246	Stage 3 CAM
3	47	0	0	IB1	VRT + PLN	SCC	Yes	PROM, Intrauterine infection	24w5d	846	277	Stage 3 CAM
4	34	0	0	IB1	VRT + PLN	SCC		PROM	19w5d	262	NA	Stage 2 CAM
5	37	0	0	IB1	VRT + PLN	SCC		Scheduled	33w6d	2294	677	No specific findings
6	40	2	0	IA2	VRT + PLN	SCC	Yes	PROM, Bleeding	23w1d	421	202	Stage 1 CAM
7	28	0	0	IB1	VRT + PLN	Verrucous		PROM	35w	NA	NA	No specific findings
8	34	0	0	IB1	VRT + PLN	SCC		PROM	34w5d	2148		Stage 1 CAM
9	32	1	0	IA2	VRT + PLN	Adenosquamous		Scheduled	34w3d	2520	650	No specific findings
10	41	0	0	IB1	VRT + PLN	SCC	Yes	Elective	33w0d	2070	500	No specific findings
11	32	0	0	IB1	VRT + PLN (during pregnancy)	SCC		PROM	34w2d	2112	518	No specific findings
12	41	0	0	IB1	VRT + PLN	SCC		Scheduled	35w5d	2126	500	No specific findings
13	39	1	1	IB1	VRT + PLN	SCC		Scheduled	35w2d	2470	510	No specific findings
14	33	0	0	IB1	VRT + PLN	Adeno		Scheduled	34w4d	3226	607	No specific findings
15	35	0	0	IB1	VRT + PLN	SCC		Scheduled	35w4d	2052	638	Microinfarction
16	31	1	0	IB1	VRT + PLN	SCC		Scheduled	35w2d	2412	522	Partial infarction
17	39	0	0	IB1	VRT + PLN	SCC	Yes	Scheduled	30w5d	1224, 1499	762	Placenta of dichorionic‐diamniotic twin
18	30	0	0	IB1	VRT + PLN (during pregnancy)	SCC		Recurrence of cervical cancer	26w6d	882	882	No specific findings
19	32	0	0	IB1	VRT (during pregnancy)	Mucinous		Scheduled	34w2d	2328	615	Partial infarction
20	34	0	0	IB1	VRT (during pregnancy)	Adeno		Scheduled	35w5d	2156	498	No specific findings
21	31	0	0	IB1	LsRT+PLN	Adeno		PROM・IUFD	19w5d	–	–	Stage 2 CAM
22	38	1	0	IB1	LsRT+PLN	Adeno		Scheduled	33w6d	1912	620	No specific findings
23	31	0	0	IB1	VRT	Mucinous		Scheduled	36w3d	2588	610	No specific findings

The V1‐V2 hyper‐variable region of the bacterial 16S rRNA gene was amplified from the extracted DNA by PCR with the modified primer pair 27Fmod (5′‐TCG TCG GCA GCG TCA GAT GTG TAT AAG AGA CAG AGR GTT TGA TYM TGG CTC AG‐3′) and 338R (5′‐GTC TCG TGG GCT CGG AGA TGT GTA TAA GAG ACA GCA TGC TGC CTC CCG TAG GAG T‐3′) with the incorporation of overhang sequences compatible with the Illumina index and sequencing adapters. To reduce bacterial DNA contamination from polymerase reagent, yeast‐made Taq DNA polymerase (Mitsui Chemicals, Chiba, Japan) was used for amplicon PCR. PCR was performed under the subtly modified condition as described in a previous study [15]. The detailed protocol is confidential and belongs to Varinos Inc., and therefore cannot be disclosed. The PCR product was purified using Agencourt AMPure XP (Beckman Coulter, Brea, CA, USA). Index attachment was performed by KAPA HiFi HotStart ReadyMix (Roche, Basel, Switzerland) with a Nextera XT Index Kit v2 (Illumina, Inc., San Diego, CA, USA). The product was purified using Agencourt AMPure XP. The final library was paired‐end sequenced at 2 × 251 bp using the MiSeq Reagent Kit v3 on the Illumina MiSeq platform (Illumina, Inc.).

### Data analysis for sequenced data

The adapter sequences were removed using trimmomatic‐0.38 [16], and the paired‐end reads were merged using EA‐Utils fastq‐join [17]. Quality control for merged sequences was performed using PRINSEQ‐lite‐0.20.4 to truncate the primer‐binding region and discard the low‐quality sequences (Q score < 25, read length <250 bp or >400 bp) [18]. Operational taxonomic units (OTUs) were created by pick_otus.py of QIIME 1.9.1 (*de novo* OTU selection using UCLUST, sequence similarity threshold = 99.5%) [19]. To assign species level taxonomy, a homology search using BLAST was conducted on the representative sequence of each OTU based on the SILVA132 [20] and STIRRUPS [21] databases. Sequences with less than 99.0% homology with the database and sequences with less than 95.0% alignment were considered chimeras and were excluded from the analysis.

The filtered sequences of each sample included sample‐derived and background contaminant bacteria. We excluded the following bacteria from the microbiome profile frequently observed in the negative control as “background bacteria”: *Burkholderia* (Unclassified), *Escherichia coli*, *Brevundimonas diminuta*, *Sphingomonas koreensis*, *Enterobacter hormaechei*, and *Methylobacterium goesingense*. We are objectively confirming contaminants using the decontam R package for these bacteria [22]. Except for *Burkholderia* (*Unclassified*), all other bacteria were also detected as contaminants by the decontam R package. While it seems certain that *Burkholderia* (*Unclassified*) is a contaminant bacterium due to its high abundance in negative controls, its detection by decontam was challenging as the OTU was universally detected in all tested samples.

Then, the resulting sequences of each sample were used for alpha and beta diversity analysis. After rarefaction analysis, including Chao1 richness and Phylogenetic Diversity (PD), alpha diversity indices were compared between groups at 3000 sequences, where the rarefaction curves of each index reached a plateau, and 15 of the 23 samples included more than 3000 sequences (Table S1). We conducted a comparison of the phylogenetic relatedness of microbial communities using beta diversity analysis in all samples. Based on OTU results, we determined UniFrac distances between samples and conducted Three‐D principal coordinate analysis. Additionally, we statistically compared the groups using PERMANOVA testing. The detection rate and the relative abundance of each bacterium were compared between groups using Fisher's exact test and Wilcoxon rank‐sum test or arcsin‐adjusted Welch's *t* test, respectively (Tables S3 and S4). We conducted a multivariate analysis where we calculated the adjusted *P*‐values for the Benjamini‐Hochberg test performed for all taxa (shown in the column labeled ‘pvalue_adjustBH’ in Tables S3 and S4), as well as an analysis for bacterial abundance using the ANCOM‐BC package (Table S5) [23].

## Results

### Details of the cases examined and histological findings of placental specimens

**Fig. 1:**
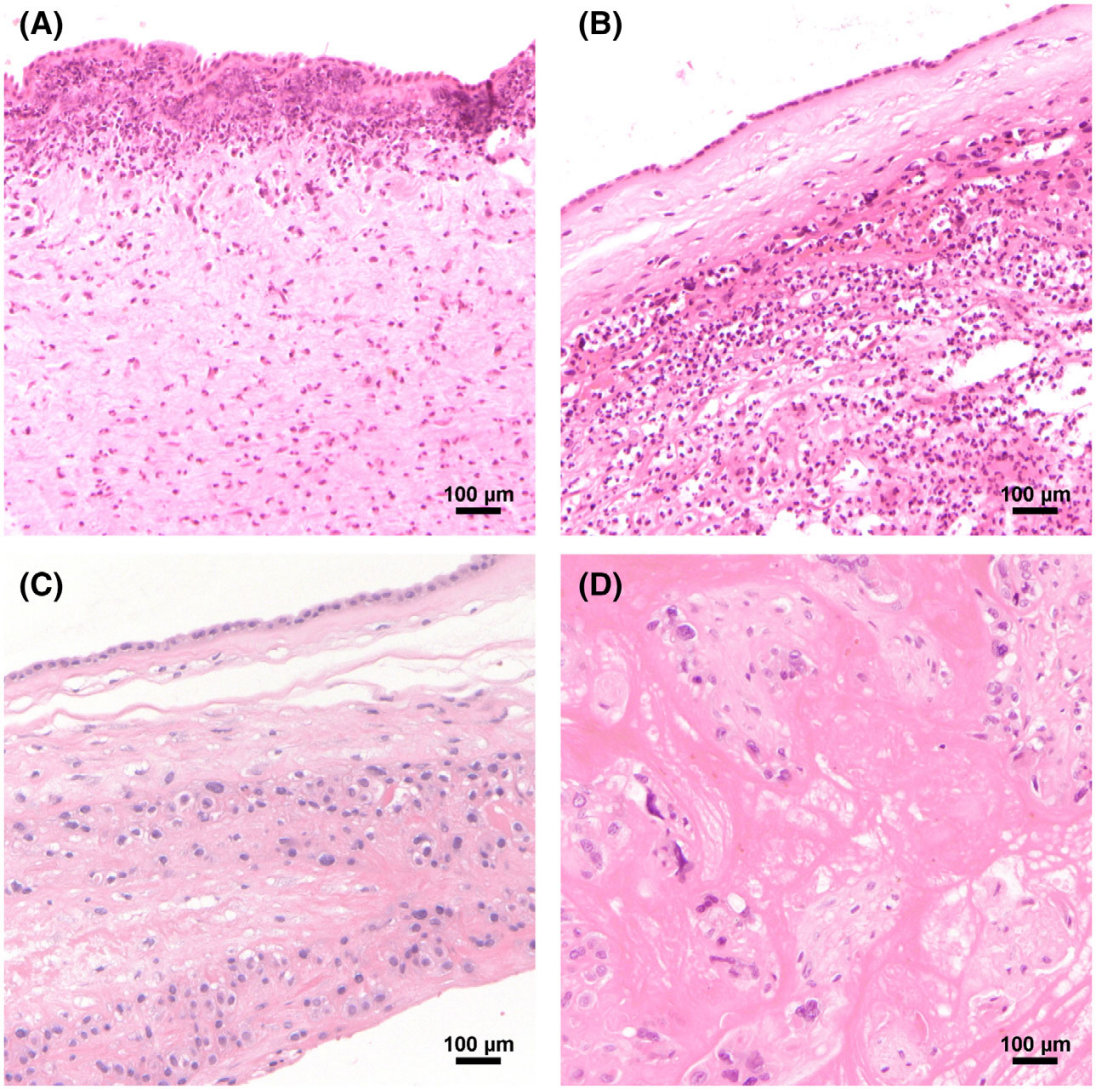
Representative histological findings from the examined placenta. (A) Stage 3 chorioamnionitis (CAM) identified in the placenta of a patient with intrauterine infection and preterm rupture of membranes (PROM; Case No. 3). Numerous neutrophils have infiltrated the amnion. (B) Stage 2 CAM observed in the placenta of a patient with PROM that resulted in spontaneous abortion at 19 weeks of gestation (Case No. 4). Neutrophil infiltration is evident in the chorion. (C) Normal chorion and amnion without CAM. PROM is also absent (Case No. 13). (D) Placental infarction in a patient with PROM but without CAM (Case No. 1). All images were obtained at 100× magnification. Scale bar, 100 μm.

The details of the cases analyzed in this study are presented in Table 1. Of these cases, 21 underwent VRT at our hospital while 2 underwent laparoscopic RT at another hospital. In addition, VRT was performed for cervical cancer detected during pregnancy in 4 cases. Almost all cases involved singleton pregnancies, although one case was a dichorionic‐diamniotic twin pregnancy. Chorioamnionitis (CAM) was determined according to Blanc's classification [24], based on histological findings of neutrophil infiltration in the amniotic membrane. Furthermore, we assessed the presence or absence of placental infarction (PI) as a positive histological finding, which is generally unrelated to infection. Examples of the histological findings are shown in Fig. 1. CAM histologies revealed the severe neutrophil infiltration extending into the chorion and/or amnion (Fig. 1A,B correspond to Case Nos. 3 and 4, respectively). Figure 1C shows the chorion and amnion without the infiltration of inflammatory cells. Figure 1D demonstrates coagulative necrosis of the placental villi, indicating PI.

### Relative bacterial abundances in each sample

**Fig. 2:**
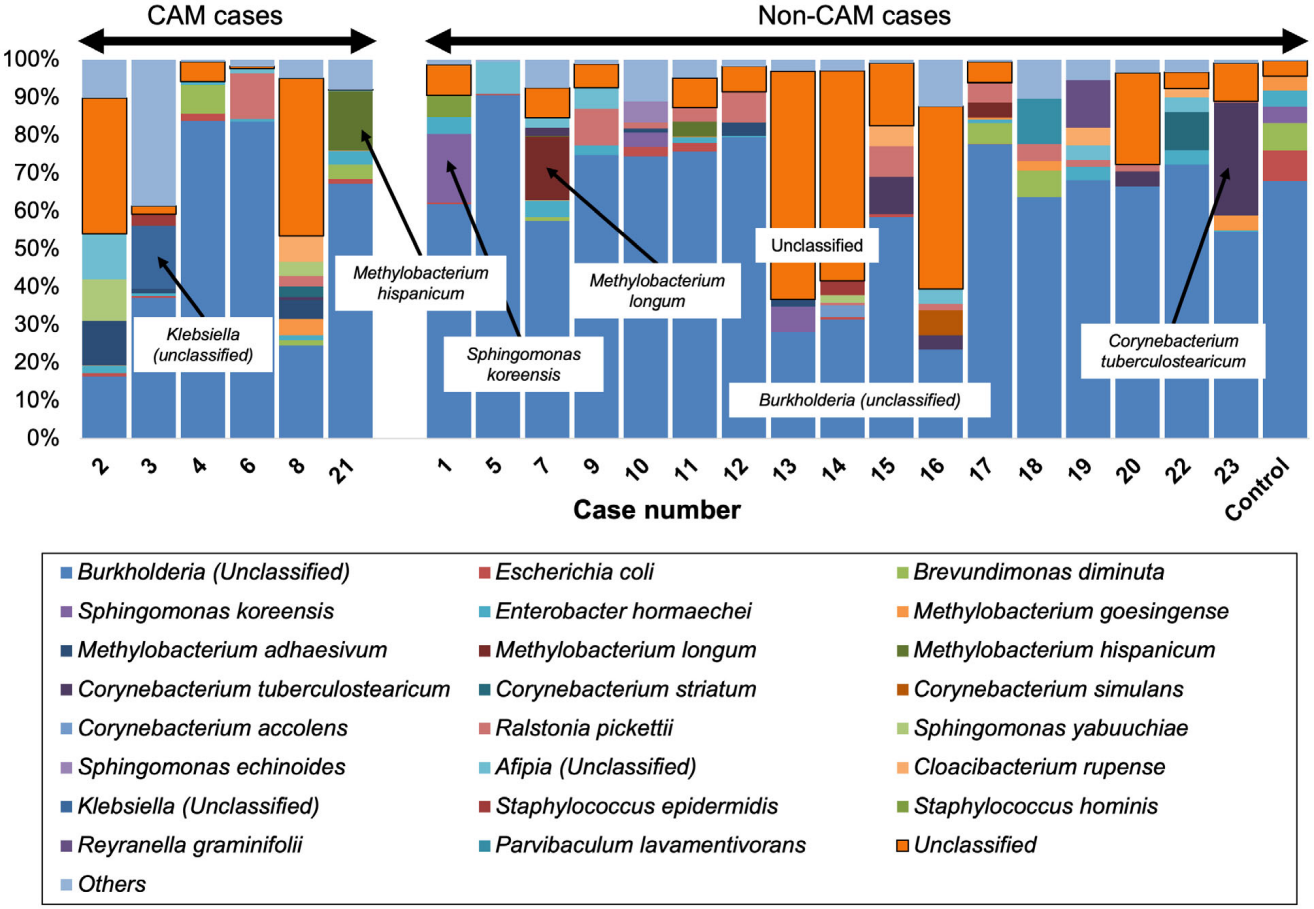
Relative abundance of the detected bacteria in placental specimens. Cases of chorioamnionitis (CAM) are presented on the left and cases of non‐CAM are shown on the right. Bacteria with a very low relative abundance (<3%) were grouped together as “Others.” Bacteria with a relative abundance of 15% or higher are indicated in the figure with arrows. In addition, “*Burkholderia* (*unclassified*)” and “*Unclassified*” were widely observed across all samples and are thus highlighted within black squares in the figure.

Figure 2 shows the relative bacterial abundance detected for each sample, categorized into CAM and non‐CAM groups. However, this abundance plot did not reveal any clear differences between the groups. We have emphasized bacteria with an abundance of 15% or higher in the figure, including *Methylobacterium* spp. and *Burkholderia* spp., which could potentially be environmental contaminants. In addition, for the sample in Case No. 3, in which *Klebsiella* spp. was detected at an abundance of 16.6%, it seemed plausible to consider it an ascending infection of Gram‐negative rods after PROM.

A more detailed bacterial taxonomy breakdown is shown in Table S2. Notably, a relatively high number of samples had “Unclassified” as the predominant result. This may reflect degradation of the extracted bacterial DNA. We conducted statistical analysis using various methods to compare the presence or absence of CAM, PROM, and PI for all detected bacteria. The bacteria showing a significant variation in the statistical testing are listed in Table 2. *Burkholderia stabilis* and *Lactobacillus crispatus* were found to have significantly higher detection rates in the non‐CAM group than in the CAM group. In addition, *Burkholderia arboris* showed a higher detection rate in the PROM group than in the non‐PROM group. *Lactobacillus crispatus* showed no significant difference between PROM and non‐PROM but tended to have a higher detection rate in the non‐PROM group. However, the relative abundance of these bacteria in all cases was extremely low, and their significance remains unclear. Furthermore, no significant differences in bacterial detection rates and abundances were observed for any of the groupings in the results of the multivariate analysis (Tables [Link], [Link], [Link]). Therefore, we expect it to be challenging to obtain stable results for the consistent identification of causative bacteria from FFPE specimens.

**Table 2:** Bacterial detection rate and abundance in each clinical condition by 16S rRNA sequencing.

Bacteria	Specimens detected (*n*)	Detection rate (%)	Mean ± SD (%)	Specimens detected (*n*)	Detection rate (%)	Mean abundance ± SD (%)	Fisher (*P*‐value)	Wilcoxon (*P*‐value)	Welch's *t* test (*P*‐value)
	Non‐CAM			CAM					
*Burkholderia stabilis*	12/18	66.67	0.19 ± 0.24	1/6	16.67	0.72 ± 1.76	1.00	0.03*	0.001**
*Lactobacillus crispatus*	8/18	44.44	0.17 ± 0.25	1/6	16.67	0.02 ± 0.04	0.61	0.21	0.03*
	Non‐PROM			PROM					
*Burkholderia arboris*	13/15	86.67	0.52 ± 0.5	8/9	88.89	3.24 ± 8.91	1.00	0.04*	0.03*
*Lactobacillus crispatus*	7/15	46.67	0.19 ± 0.27	2/9	22.22	0.03 ± 0.07	0.39	0.20	0.07

*P < 0.05, **P < 0.01.

### Comparison of bacterial diversity in each clinical situation

**Fig. 3:**
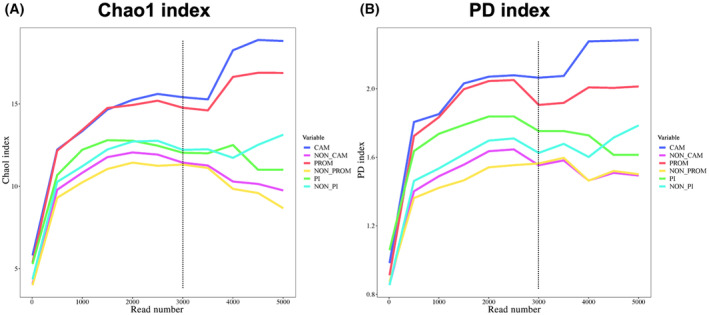
Alpha rarefaction curves of diversity indices (Chao1 index and PD index). The alpha rarefaction curve visualizes a diversity index that changes with the number of randomly extracted sequence reads. As shown in the figure, when the number of reads on the x‐axis exceeded 3000 (dashed line), a variability in the diversity index occurred in each group (A: Chao1 index, B: PD index). Therefore, in this study, we calculated the diversity index under the condition of 3000 reads.

We analyzed the alpha diversity to assess the microbial community diversity of FFPE placenta. This analysis compared the presence or absence of CAM, PROM, and PI, similar to the investigation of microbial detection rates. In the examination of alpha diversity, we had already removed sequence reads originating from background bacteria in environmental sources and reagents. The average eligible sequence read count per sample after background removal were relatively low at 4130 ± 3164.3 and showed substantial variability (Table S1). Therefore, we conducted preliminary analysis using alpha rarefaction analysis to observe the changes in alpha diversity indices (Chao1 and PD) in relation to varying sequence read counts. The results indicated that the diversity indices in each grouping plateaued at approximately 3000 sequence reads, with subsequent variations observed (Fig. 3A,B). Therefore, in this study, we set 3000 sequence reads as the requirement for alpha diversity assessment.

**Fig. 4:**
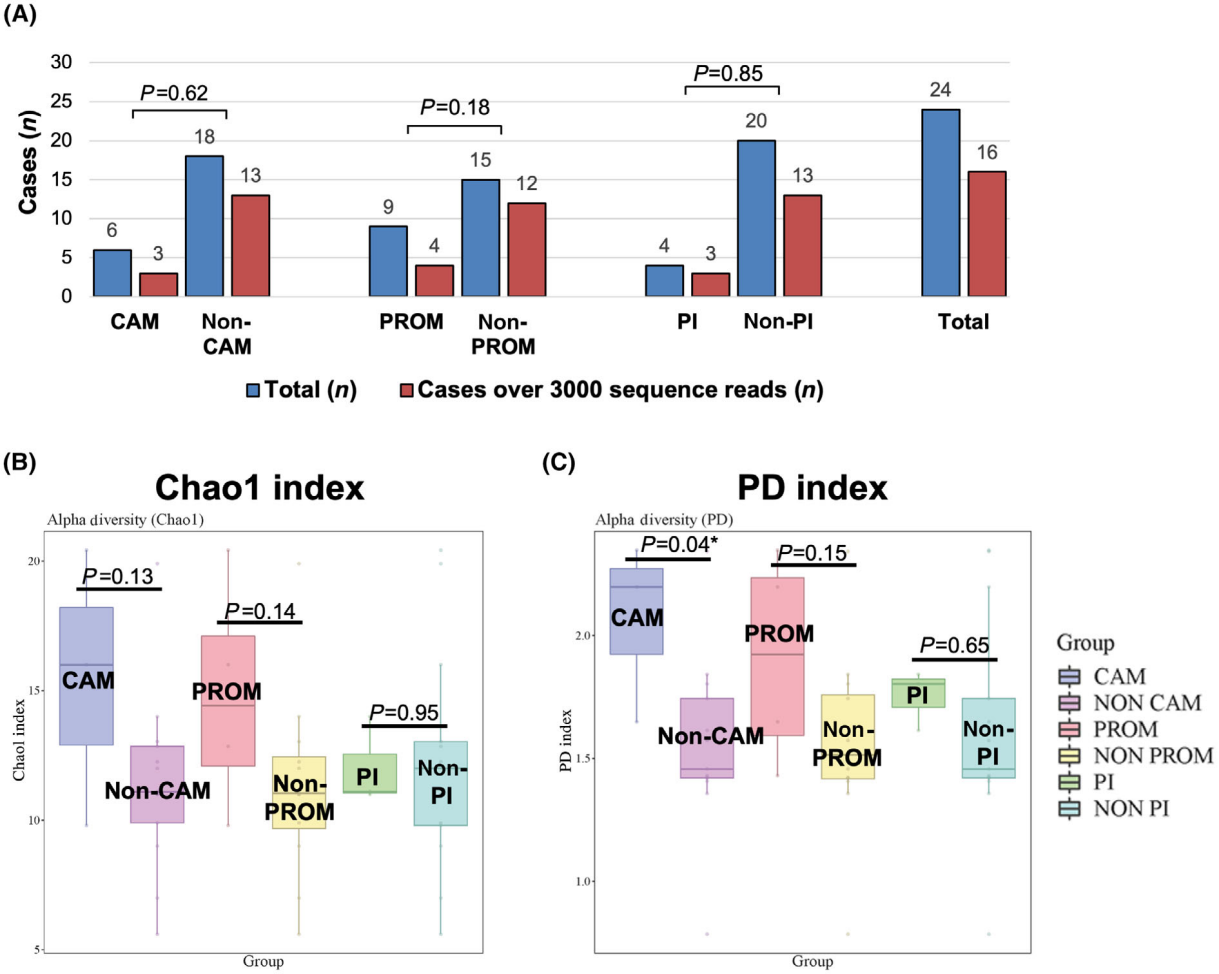
Alpha diversity analysis of each clinical condition (Chao1 index and PD index). The bar graph shows the total number of samples on the left and the number of samples that still retained over 3000 reads after the elimination of background bacteria on the right. Group comparisons were statistically assessed using the chi‐square test (A). The calculated alpha diversity measures for each sample were aggregated and subjected to statistical analysis. The Chao1 index (B) is displayed on the left while the PD index (C) is presented on the right. Error bars represent the standard deviation, and statistical analysis was conducted using Student's *t*‐tests (*P < 0.05). The indices in the CAM and PROM groups tended to be higher than in the non‐CAM and non‐PROM group. Notably, when the PD index was compared between CAM and non‐CAM groups, a statistically significant difference was observed (*P* = 0.04; *Statistically significant).

The number of samples used for the analysis is shown in Fig. 4A. There were no missing values in any of the groups, and no significant differences in sample numbers were observed among the groups. The results of the analysis of Chao1 and PD indices are shown in Fig 4B and C, respectively. While the Chao1 index tended to be higher in the CAM and PROM groups, no significant differences were noted. On the other hand, the PD index showed a significantly higher result in the CAM group (*P* = 0.04), suggesting a potentially higher microbial diversity in CAM cases. Regarding PI, there was no discernible trend in index levels between the PI and non‐PI groups. We also investigated beta diversity using the same grouping (Fig. S1). However, no significant differences were observed in any of the comparisons between microbial communities.

## Discussion

While genomic analysis using FFPE tumor specimens has become commonplace for cancer genome profiling, few reports have demonstrated the usefulness of microbiome analysis [13]. We previously demonstrated the retrospective detection of invasive *Streptococcus pyogenes* infection using the same method used in this study [14]. However, in the present study, no cases were observed where a clear identification of the causative agent was possible. The results suggest that background contamination had a significant impact on microbiome analysis.

A comparison study between FFPE and frozen fetal membrane specimens suggested that FFPE‐derived samples often contain a higher amount of bacterial DNA originating from the paraffin wax or DNA extraction reagents [25]. Attempts have been made to study cancer‐specific microbiomes using FFPE specimens obtained from patients with colorectal or breast cancer [26, 27, 28]. While contamination is a concern, these reports suggest that such studies can feasibly be conducted using FFPE samples. To use FFPE specimens for microbiome analysis, research will be required to identify the optimal protocol for specimen handling, similar to what has been done for cancer genome profiling, considering factors such as storage duration and reagent contamination. On the other hand, the FFPE‐based methods for confirming the causative bacteria in invasive infections have already been reported to be effective in many reports [14, 29, 30, 31]. However, regarding *Ureaplasma* spp., well known for its causative association with PROM [32, 33, 34], it was not detected in samples from either PROM or non‐PROM cases in this study. Therefore, the possibility cannot be ruled out that CAM and PROM cases following RT may tend to form a specific bacterial community due to the large cervical defect. Further investigation using fresh or frozen perinatal specimens is desired for future studies.

In general, *Lactobacillus* spp. predominating in the vaginal microbiota is considered healthy [35]. Any imbalance in this microbiome can lead to bacterial vaginosis. In the presence of *Lactobacillus* spp., the vagina is maintained at a lower environmental pH, which has a sterilizing effect [36]. Therefore, when the prevalence of *Lactobacillus* spp. decreases, it creates a favorable environment for the proliferation of various miscellaneous bacteria, including *Anaerococcus*, *Peptoniphilus*, *Prevotella*, and *Streptococcus*, resulting in an increased bacterial diversity [37]. In our study, particularly in patients histologically diagnosed with CAM, there was a higher alpha diversity of bacteria. Reports have indicated that the bacterial alpha diversity of amniotic fluid samples fluctuates with CAM severity [38]. According to their findings, bacterial selection occurs due to the dominance of the causative bacteria in stage 3 CAM, leading to a decrease in alpha diversity. However, prior to that stage, the alpha diversity increased with an increase in the CAM stage. This aligns with our results and is not contradictory in any way. In terms of the abundance of the detected bacteria in our study, a large abundance of water‐related environmental bacteria were found, such as *Burkholderia* spp. [39] and *Methylobacterium* spp. [40], which raise suspicions of contamination and appear to undermine the results. Considering the findings of the alpha diversity analysis in this study, it is still believed that an intrauterine infection can be detected in FFPE placental specimens. The tendency for higher bacterial diversity in CAM and PROM cases, as well as the significant observation of a tendency for higher levels of *Lactobacillus crispatus* in the non‐CAM, non‐PROM group despite its lower abundance, was deemed noteworthy. Therefore, it is once again emphasized that the proper management of the genital microbiome is crucial for preventing unexpected preterm births associated with intrauterine infection after RT.

Several methods are currently suggested for improving the vaginal flora. One such method is lactoferrin, which acts as a prebiotic [41, 42, 43]. It is thought to contribute to the treatment of refractory bacterial vaginosis and the normalization of intrauterine bacterial flora [44]. Preparations containing lactoferrin are commercially available in various forms as a supplement, making it an easily accessible option. Another approach is probiotic therapy through the transplantation of *Lactobacillus* spp. into the vagina. While there are various reports on this method, the most promising involves the direct transplantation of strains of *Lactobacillus crispatus* CTV‐05 (Lactin‐V) [45]. Surprisingly, CTV‐05 treatment resulted in sustained colonization of *Lactobacillus* spp. in 79% of patients after treatment and effectively prevented recurrence. Another recent development is the use of vaginal microbiota transplantation. This method involves the transplantation of the vaginal microbiota from a woman with a healthy bacterial flora directly into another individual and has been shown to be highly effective in cases of recurrent bacterial vaginosis [46, 47, 48]. In the present study, CAM was found to be associated with PROM after RT at a high frequency (6 out of 9 cases) and may be related to vaginal bacterial dysbiosis. Therefore, efforts to improve the vaginal flora should be routinely considered for pregnancy after RT.

A limitation of this study is the insufficient number of specimens. Radical trachelectomy is not a frequently performed surgery for the treatment of cervical cancer, limiting the analysis to 23 cases. One of our main goals was to identify bacterial pathogens characteristic of intrauterine infection following radical trachelectomy, but this was not elucidated in the present analysis. Nonetheless, the significant increase in alpha diversity observed in the CAM group clearly indicates an association between CAM and intrauterine infection following radical trachelectomy, suggesting that the true causative bacterial pathogens may be included in the OTU analysis results. Additionally, it was shown that *Lactobacillus crispatus* and *Burkholderia stabilis* were more prevalent in the non‐CAM group, suggesting they may have a protective role against CAM. However, caution is required when interpreting these results of identified bacteria. Since the statistical OTU analysis presented is based on non‐adjusted *P*‐values, the significance level is lenient, and the reliability is compromised compared to multivariate analysis. Therefore, the findings presented in this paper should be considered as merely indicative of a trend. Unfortunately, multivariate analysis did not identify pathogens independently associated with CAM or PROM. Since metagenomic analysis involves a large number of bacterial species, multivariate analysis may be unsuitable for studies using relatively rare specimens like the present study. Further studies using more samples are desirable. Furthermore, while metagenomic analysis using FFPE placental specimens showed significant limitations, we were able to confirm the statistically significant presence of placental dysbiosis in CAM cases. For the management of high‐risk cases following RT, maintenance of a healthy vaginal microbiome is considered crucial.

## Conflict of interest

The authors declare that they have no conflict of interest. All authors have contributed significantly to the research described in the paper and have reviewed the manuscript and agree with its contents and with the authorship listed.

### Peer review

The peer review history for this article is available at https://www.webofscience.com/api/gateway/wos/peer-review/10.1002/2211-5463.13892.

## Author contributions

RT is the principal author and the main contributor to writing the manuscript. TM designed the research and performed sample collection and data analysis. MU, SO, WA, ST, KA, and YS performed detailed analysis of pathogens using next‐generation sequencing. TK performed detailed histological examination of placental specimens. TB, TE, and SI treated patients and collected clinical data. TS supervised this study and corrected the manuscript. All authors read and approved the final manuscript.

## Supporting information


**Fig. S1.** Beta diversity analysis of each clinical condition.

**Table S1.** DNA library concentration and number of sequencing reads for each FFPE specimen.

**Table S2.** Detailed abundance of the detected bacteria in each sample of FFPE placenta.

**Table S3.** Statistical analysis of the association between the presence of each bacterial species and CAM.

**Table S4.** Statistical analysis of the association between the presence of each bacterial species and PROM.

**Table S5.** Statistical comparison of the composition of microbiomes using ANCOM‐BC package.

## Data Availability

The data that support the findings of this study are available from the corresponding author upon reasonable request.
