# Supplementary figures and images for: Microbiological investigation of pregnancies following vaginal radical trachelectomy using 16S rRNA sequencing of FFPE placental specimens
Source: FEBS Open Bio. 2024 Sep 8;14(11):1825–36. doi: 10.1002/2211-5463.13892 (PMC11532974; doi:10.1002/2211-5463.13892)

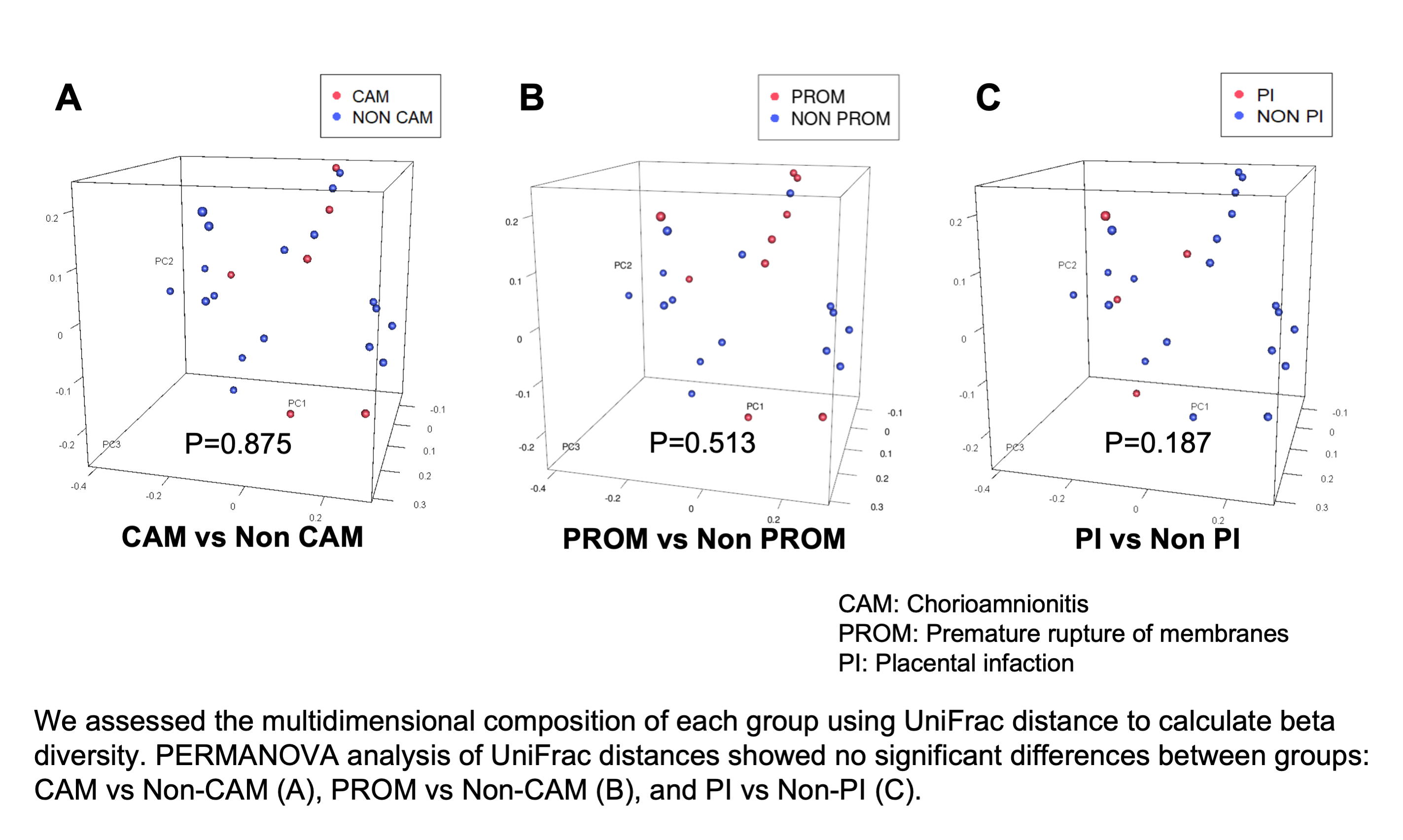

Supplement: Supplementary file 1 — Fig. S1. Beta diversity analysis of each clinical condition. [file FEB4-14-1825-s004.tiff]
